# ECO: the Evidence and Conclusion Ontology, an update for 2022

**DOI:** 10.1093/nar/gkab1025

**Published:** 2021-11-19

**Authors:** Suvarna Nadendla, Rebecca Jackson, James Munro, Federica Quaglia, Bálint Mészáros, Dustin Olley, Elizabeth T Hobbs, Stephen M Goralski, Marcus Chibucos, Christopher John Mungall, Silvio C E Tosatto, Ivan Erill, Michelle G Giglio

**Affiliations:** Institute for Genome Sciences, University of Maryland School of Medicine, Baltimore, Maryland, USA; Institute for Genome Sciences, University of Maryland School of Medicine, Baltimore, Maryland, USA; Institute for Genome Sciences, University of Maryland School of Medicine, Baltimore, Maryland, USA; Institute of Biomembranes, Bioenergetics and Molecular Biotechnologies, National Research Council (CNR-IBIOM), Bari, Italy; Department of Biomedical Sciences, University of Padova, Padova, Italy; Structural and Computational Biology Unit, European Molecular Biology Laboratory, Heidelberg 69117, Germany; Institute for Genome Sciences, University of Maryland School of Medicine, Baltimore, Maryland, USA; Department of Biological Sciences, University of Maryland Baltimore County, Baltimore, Maryland, United States; Department of Biological Sciences, University of Maryland Baltimore County, Baltimore, Maryland, United States; Institute for Genome Sciences, University of Maryland School of Medicine, Baltimore, Maryland, USA; Division of Environmental Genomics and Systems Biology, Lawrence Berkeley National Lab, Berkeley, California, USA; Department of Biomedical Sciences, University of Padova, Padova, Italy; Department of Biological Sciences, University of Maryland Baltimore County, Baltimore, Maryland, United States; Institute for Genome Sciences, University of Maryland School of Medicine, Baltimore, Maryland, USA

## Abstract

The Evidence and Conclusion Ontology (ECO) is a community resource that provides an ontology of terms used to capture the type of evidence that supports biomedical annotations and assertions. Consistent capture of evidence information with ECO allows tracking of annotation provenance, establishment of quality control measures, and evidence-based data mining. ECO is in use by dozens of data repositories and resources with both specific and general areas of focus. ECO is continually being expanded and enhanced in response to user requests as well as our aim to adhere to community best-practices for ontology development. The ECO support team engages in multiple collaborations with other ontologies and annotating groups. Here we report on recent updates to the ECO ontology itself as well as associated resources that are available through this project. ECO project products are freely available for download from the project website (https://evidenceontology.org/) and GitHub (https://github.com/evidenceontology/evidenceontology). ECO is released into the public domain under a CC0 1.0 Universal license.

## INTRODUCTION

Biocuration is the act of asserting that a particular piece of information is relevant to, or describes an aspect of, a biological entity (1). The documentation of such annotations provides not only a repository of knowledge but also forms a foundation that is used to inform further research. Significant effort is invested in designing data models and tools for the effective storage of annotations attached to species, genes, proteins and other biological entities. The amount of information available about biological entities and systems continues to expand at unprecedented rates due to the continued development of multiple high-throughput data generation techniques. It is vital to have robust systems for the effective capture and redistribution of annotation information resulting from biocuration processes in order to facilitate mining of this information for analysis and hypothesis generation. Ontologies and controlled vocabularies are an efficient way to capture annotation information in a computable format that facilitates its downstream use (1).

**Figure 1.**
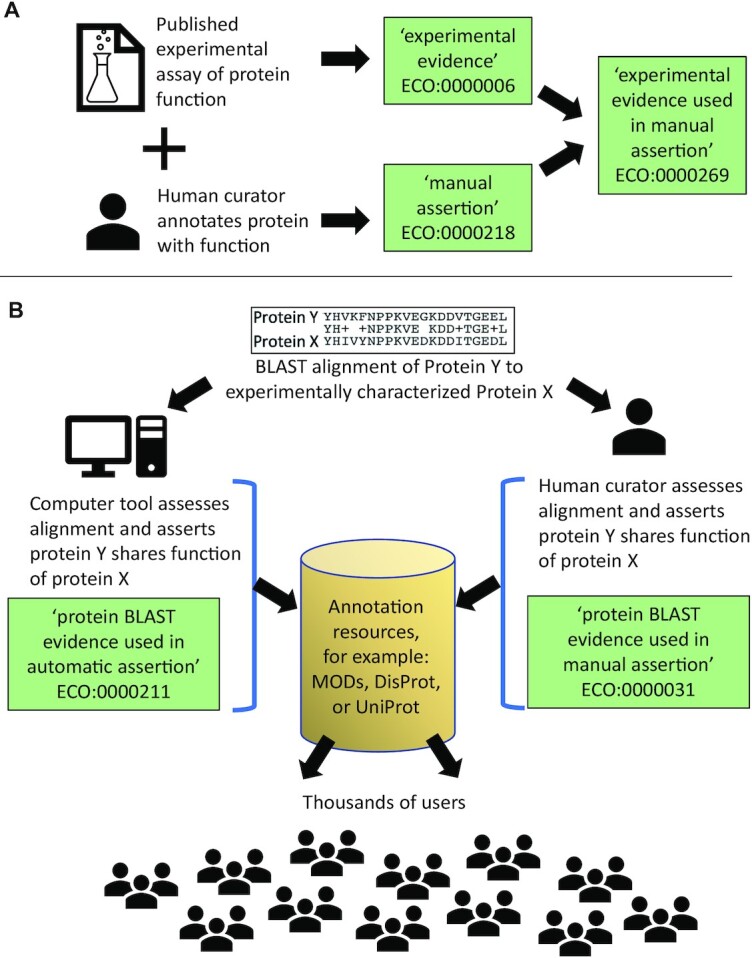
ECO assertion types and example annotations. (**A**) example of how evidence type and assertion type are combined to make leaf nodes describing both. (**B**) Illustration of the use of sequence alignment for both an automatic and manual annotation, the entry of that information into a database, and the distribution of that information to the user community. Green boxes contain ECO terms. MOD = model organism database.

Biological annotations consist of at least three parts: the object about which information is being asserted (e.g. a protein), the aspect of the object to be asserted (e.g. a function), and the evidence that supports the assertion (e.g. evidence from an enzymatic assay). The documentation of evidence provides the scientific basis on which the assertion is built and is therefore a key component of the annotation (Figure 1). Evidence can be of many types including sequence analyses, curator inferences or results of laboratory experiments (Figure 1). The Evidence and Conclusion Ontology (ECO) is used to capture the types of evidence that support biological assertions (2). Consistent capture of evidence information allows tracking of annotation provenance, establishment of quality control measures, and evidence-based data mining.

ECO is used in the annotation and biocuration processes of dozens of database resources. These resources include model organism databases as well as databases focused on particular topic areas (e.g. CollecTF (bacterial transcription factors) (3), DisProt (intrinsically disordered proteins) (4)). In addition, ECO is used by two of the most prominent resources in biology: UniProt (5) and the Gene Ontology Consortium (6). As a point of reference, within UniProtKB, 98.9% of proteins in Swiss-Prot and 99.9% of proteins in TrEMBL have annotations employing ECO terms. A full list of groups using ECO can be found on the ECO website (https://evidenceontology.org/). Through the databases that employ ECO, thousands of individual users interact with, and benefit from, annotations using ECO (Figure 1). Here we describe recent developments in ECO and its associated resources since our last update (2)

## ECO STRUCTURE AND CURRENT STATUS

**Figure 2.**
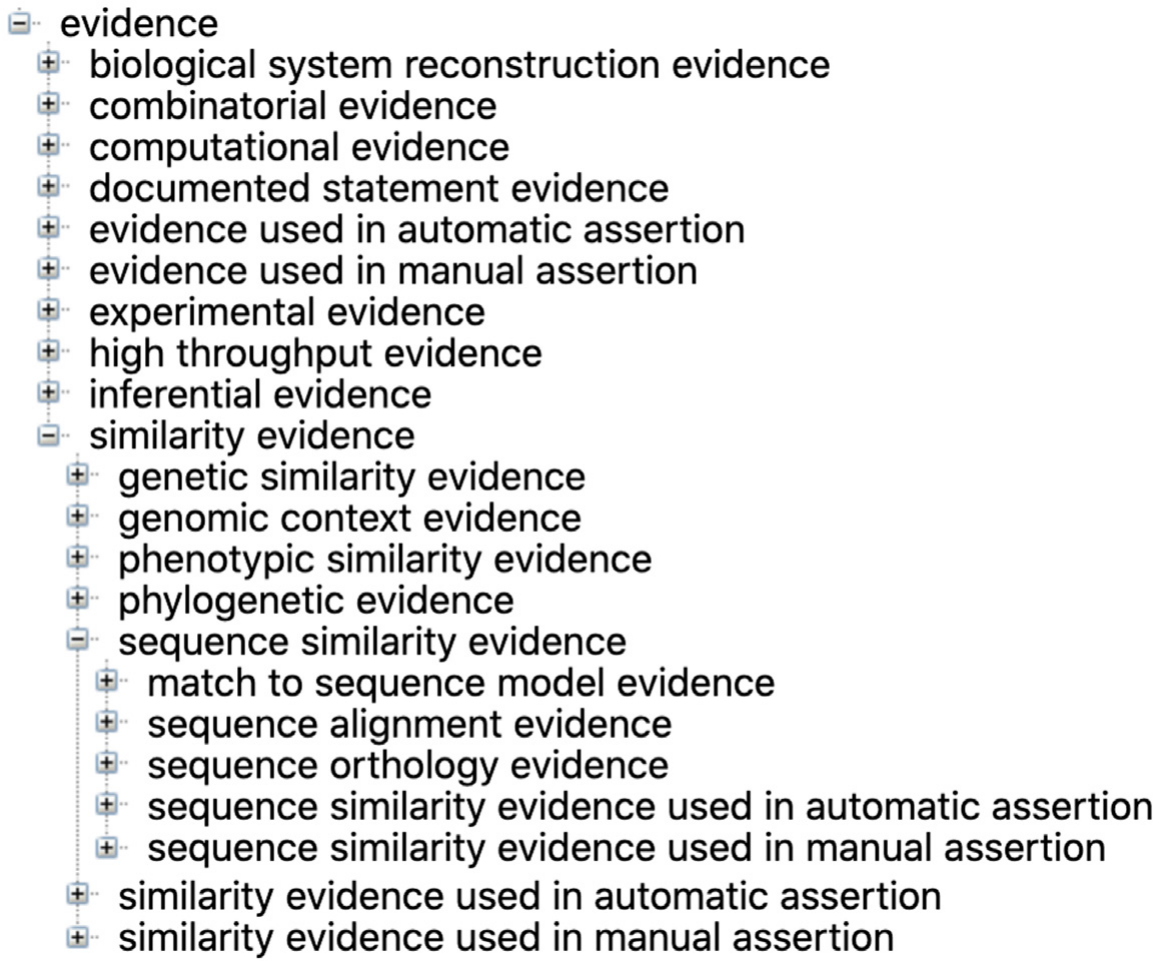
Tree view of ECO. This tree shows all of the top level children of ‘evidence’ as well as a sample of more granular terms under ‘similarity evidence’ including assertion-type leaf nodes. Tree made using the BioPortal tool (7).

As described previously (2), ECO is structured along two primary root nodes: ‘evidence’ and ‘assertion method’. The ‘evidence’ node is the overall parent for terms describing types of evidence and contains classes such as ‘similarity evidence’ (ECO:0000041) and ‘experimental evidence’ (ECO:0000006). More granular children provide classes for very specific evidence types including ‘protein BLAST evidence’ (ECO:0000208), and ‘loss-of-function mutant phenotype evidence’ (ECO:0000016). The assertion method branch captures whether the assertion has been made using an automated computer curation process or by a human curator who has manually reviewed the evidence information to inform the assertion. There are only two terms in the ‘assertion method’ branch: ‘automatic assertion’ and ‘manual assertion’. Every evidence-type term in the ‘evidence’ branch of ECO has two children representing the two possible assertion types that could be used with that evidence type (Figure 1). These are constructed with logical axioms using the ‘used_in’ relationship. For example, a child of ‘protein BLAST evidence’ (ECO:0000208) is ‘protein BLAST evidence used in automatic assertion’ (ECO:0000211) which has the logical axiom ‘protein BLAST evidence and *used_in* some automatic assertion.’ Under this structure, all leaf nodes in ECO are terms that combine a type of evidence and an assertion method and all evidence-type terms have both an automated and manual assertion type child term. Figure 2 shows a tree generated using the BioPortal tool (7) that includes the upper levels of the ‘evidence’ branch with selected branches expanded to see both automated and manual assertion child terms. We have established this structure for logical consistency, even though not all evidence types have yet been used for automated curation. In addition to the logical axioms underlying the automatic and manual assertion terms, we also strive to provide logical definitions for ECO terms that link them to other ontologies such as the Gene Ontology GO (6) and the Ontology for Biomedical Investigations (OBI) (8). Currently, there are 1941 terms in ECO, up from 1515 in our last report.

## ECO TERM DEVELOPMENT

ECO is developed in the Web Ontology Language (OWL) using Protégé (9) for viewing and small scale edits and ROBOT (10) for larger-scale modifications and releases. Term development in ECO is driven primarily by user requests. We employ a GitHub issue tracker (https://github.com/evidenceontology/evidenceontology/issues) where users can submit requests for new terms or changes to existing terms. We also use this tracker to organize the ECO team's ongoing development efforts. Often we find that a request from a user will alert us to regions of the ontology that can use further refinement or development and we add this to our development queue. Described below are some of the more significant areas of term development since our last update on ECO (2).

### DisProt

One of the newest users of ECO is the DisProt database (4). DisProt is focused on intrinsically disordered proteins. Previously, it was thought that proteins must achieve a stable 3D structure in order to function. However, in the past two decades it has become clear that many proteins, called intrinsically disordered proteins (IDPs), fulfill their often pivotal biological roles while in a partially or fully dynamic state, without a fixed 3D structure (11). Several IDPs are able to change their structural properties in response to interaction with binding partners, changes in proteoform or changes in environmental conditions (12,13). It is predicted that as many as 40% of eukaryotic proteins contain disordered regions (14), and due to their important biological roles in signaling, regulation and stress response, many of these proteins are implicated in disease. It is now clear that this class of proteins have important and unique characteristics and functions and the study of IDPs has become a highly active area of research (13).

DisProt is a manually curated resource that captures annotations of IDPs and intrinsically disordered regions (IDRs) of proteins from literature (4). Curators at DisProt engage in primary annotation of the literature to gather information about IDPs and IDRs. Part of this process is the capture of evidence associated with the structural determination of the proteins. Until recently, to meet this need, DisProt was using a combination of in-house terms and terms from the Protein Standards Initiative Molecular Interactions (PSI-MI) vocabulary for protein–protein interactions (https://www.psidev.info/groups/molecular-interactions) (15). In an effort to improve the consistency of evidence annotations, DisProt chose to shift to the use of ECO for evidence capture. Although ECO had some of the terms needed by DisProt for annotation, there were many types of evidence that were not yet captured as ECO terms. Therefore, the ECO team engaged in a collaboration with DisProt curators to expand ECO in areas relevant to the study of IDPs and IDRs.

The collaboration process involved DisProt curators proposing new term labels, definitions and parentage. ECO team members reviewed the suggestions and back-and-forth discussions between DisProt and ECO produced a final set of terms. The DisProt-ECO collaboration resulted in new terms in multiple branches of ECO. While many of the terms were in areas related to microscopy (e.g. ‘fluorescence microscopy evidence’ (ECO:0006271)), spectroscopy (e.g. ‘Fourier-transform infrared spectroscopy evidence’, (ECO:0006191)), and crystallography (e.g. ‘X-ray crystallography-based structural model with high relative B-factor values’, (ECO:0006188)), there were also terms developed for experimental areas related to physical interactions (e.g. ‘microscale thermophoresis evidence’ (ECO:0006261)) and binding (e.g. ‘competitive binding evidence’ (ECO:0006264)). In fact, multiple new branches relevant for the IDP field were created all over ECO, e.g. ‘circular dichroism evidence’ (ECO:0006177) and ‘cryogenic electron microscopy evidence’ (ECO:0006181), with these new branches often including new child terms of their own. Additional new terms in the direct assay branch of experimental evidence include ‘selective antibody-based structural conformation evidence’ (ECO:0006194) and ‘heat capacity-based evidence’ (ECO:0006192). Of note, not only the experimental branch of ECO received new terms; the new term ‘author inference’ (ECO:0006185) was also created as a child term of ‘author statement’ (ECO:0000204) to better capture the nuances of the source of information used to make assertions. In total, 48 new ECO terms were created as part of the DisProt project. It is important to note that several of the newly created terms describe evidence from experimental techniques widely used in several other fields of biology apart from IDP research and therefore will be of broad utility. Furthermore, investigation of ECO for the creation of the DisProt terms revealed the need for revisions to some existing ECO terms and relationships thus improving ECO as a whole. We look forward to continued collaboration with the DisProt team as they continue their work and identify needs for additional evidence terms.

### Reorganization of, and addition to, upper level ECO classes

In response to a variety of requests by users, we have reorganized some of the first-level children of ‘evidence’, one of the two root nodes of ECO (Figure 2).

#### Documented statement evidence

A previous first-level child term ‘author statement’ (ECO:0000204) was moved under a newly created first-level child term called ‘documented statement evidence’ (ECO:0006151). The new term has additional child terms for statements from individuals such as ‘medical practitioner statement evidence’ (ECO:0006152) and ‘self-reported patient statement evidence’ (ECO:0006154).

#### Computational evidence

Another new first-level child of ‘evidence’ is ‘computational evidence’ (ECO:0007672). This term was created in part to meet the needs for generation of terms involving combinatorial evidence and in part to be the counterpart of ‘experimental evidence’. We are still in the process of identifying ECO terms that should be children of ‘computational evidence’. Towards that goal, we have added ‘computational evidence’ parentage to the ‘sequence similarity evidence’ (ECO:0000044) branch.

#### Genomic context evidence

The term ‘*genomic context evidence’* (ECO:0000177) was previously a first-level child of ‘evidence’. We have recategorized that branch as a child of ‘similarity evidence’ (ECO:0000041). Our review of the terms under ‘genomic context evidence’ indicated that they involved comparing one thing to a related thing in some way, generally involving similarity of function, content, sequence or some combination of those.

### Review of ECO structure

Part of our continued development of ECO involves inspecting the ontology for terms or branches that are redundant, confusing, or misplaced. Here we describe two selected examples of areas where changes were made.

#### Mutant phenotype evidence

Previously, there were terms representing types of evidence related to mutant phenotypes that were not children of ‘mutant phenotype evidence’ (ECO:0000015), but were instead located in other areas. This prompted a review of all terms related to mutant phenotypes. Terms were collected and reviewed, and then revised and rehomed, as appropriate, under ‘mutant phenotype evidence’.

#### Protein assay evidence

The term ‘protein assay evidence’ (ECO:0000039) was a grouping term that had many child terms with second parents in other parts of ECO. Those second parents provided more meaningful information about the type of evidence involved. The ‘protein assay evidence’ term was vaguely defined and allowed the grouping of any type of evidence in which a protein was involved, often grouping terms from very different evidence contexts, for example ‘enzymatic activity assay evidence’ (ECO:0000005) and ‘immunoprecipitation evidence’ (ECO:0000085). The term ‘protein assay evidence’ was deprecated. Any children that didn’t already have second parents were rehomed.

### New terms for long-term ECO user groups

We continue to receive requests for new terms and other term-related changes from groups with whom we have collaborated for many years. Examples of the groups for which we’ve added multiple terms since our last ECO update (2) are: the Ontology of Microbial Phenotypes (16), CACAO (17), CollecTF (3), the Planarian Anatomy Ontology (18) and Bgee (19).

## ADDITIONAL PROJECT RESOURCES FOR ECO USERS

In addition to the ontology itself, the ECO project has produced additional resources of help to users and developers. These include mapping files and annotated datasets. This section describes recent efforts in this area.

### New ECO-to-GO evidence code mapping process

Gene Ontology (GO) evidence codes (6) were a significant portion of the terms that made up the original versions of ECO and there remains a very tight collaboration between the two projects. All of the GO evidence codes have representation within ECO, generally corresponding to high-level ECO terms. When expansions to the GO evidence codes are needed, GO curators work with ECO on their development so that the evidence codes have corresponding terms in ECO. Many of the model organism databases that contribute to the GO annotation repository use native ECO terms for their annotations, thus making use of the full extent of more granular terms available in ECO. However, to submit data to the GO repository in the GO Annotation File (GAF) format, these more granular ECO terms must be mapped to the more general ECO terms that correspond directly to GO evidence codes. As previously described (2), in order to facilitate this process, ECO provides two files:

- gaf-eco-mappings.txt - This file provides mappings of GO evidence codes, or particular combinations of GO evidence codes and ‘GO_references’ (citable abstracts describing scientific methods), to ECO term ids. These are meant to be equivalence mappings.
- gaf-eco-mapping-derived.txt - This file provides mappings of the more granular ECO terms to the GO evidence codes. This file allows anyone who uses the full ECO vocabulary to be able to convert their evidence information to a format required by the GO and associated tools.

Previously, the gaf-eco-mapping-derived.txt file was generated automatically by traversing the ECO graph to collect all descendent terms for each of the ECO terms in the equivalence mappings in the gaf-eco-mappings.txt file. However, we have found that as we continue to develop ECO, some of the more granular ECO terms have been positioned in the ontology such that they are no longer descendants of the same high-level ECO terms from the ECO-GO-evidence-code equivalence pairs. This is indicative of the fact that in some situations, GO groups evidence types under one evidence code that are located in different branches within ECO. This occasionally resulted in granular native ECO annotations mapping to the incorrect GO evidence code.

To address this challenge, we have changed the way we generate the gaf-eco-mappings-derived.txt file to allow more flexibility and provide for curation of mappings at the level of every ECO term. This was accomplished through the addition of the ‘has GO evidence code’ annotation property field for ECO terms. To provide initial values for this field for existing terms, we used the last automatically generated version of the gaf-eco-mappings-derived.txt file. Moving forward, populating this field for new ECO terms will be a required part of the term-creation process. All new versions of the gaf-eco-mappings-derived.txt file will now be generated from the values associated with each term in the ontology. This gives us the freedom to move granular terms around to fit the design patterns of ECO, while still retaining the ability to provide correct mappings for GO users by curation of the ‘has GO evidence code’ field. The shift to the new process for generating the gaf-eco-mappings-derived.txt file started as of the September 2021 ECO release.

### CollecTF annotated corpus

The CollecTF data resource captures information about bacterial transcription factors, the DNA sequences they target, and the genes they regulate through curation of published experimental results. These annotations are integrated into resources such as UniProt and the GO annotation repository (20). As described in our last update (2), we have been collaborating with CollecTF in multiple areas. Most recently, we have worked with CollecTF curators to manually assign ECO terms to sentences from a selection of published papers. Through this process, we established a set of annotation guidelines that were then employed by undergraduate students to annotate a collection of 84 publications on bacterial transcription factors. This resulted in the ECO-CollecTF corpus, a freely available set of sentence-level annotations of ECO terms to text (21). Such a corpus can be used for text mining and other machine learning operations. To our knowledge, this is the only corpus that provides annotations focused on the capture of evidence statements in text. The corpus has also provided a source for ‘example of usage’ annotations for a subset of ECO terms. Usage examples are intended to assist annotators in determining the appropriateness of an ECO term by reviewing its actual use in scientific articles. In total, 63 usage examples were populated for 45 ECO terms (21). The relatively low number of ECO terms for which we established new usage examples is a reflection of the narrow biological scope of the chosen papers (bacterial transcription factors) and the fact that we only selected the annotations with the highest inter-annotator agreements to be usage examples. In defining the ECO-CollecTF corpus, we also explored the accessibility and longevity issues faced by corpora. To address corpus obsolescence, we proposed and demonstrated the embedding of corpora within the referenced ontologies, releasing a version of ECO incorporating the ECO-CollecTF corpus (http://ceur-ws.org/Vol-2807/paperF.pdf).

### SRI evidence term mappings

SRI International (formerly Stanford Research Institute) maintains an evidence vocabulary used for their work (22**)**. At the request of an ECO user, we created a mapping between ECO and the SRI system. There are 48 terms in the SRI evidence vocabulary as of September 2021. We mapped these to 27 unique ECO terms. During the process of creating the mappings, we identified several specific SRI terms for which there was no correspondingly granular ECO term. These are being explored as areas for potential ECO term expansion. The ECO-SRI mapping file is available from the ECO website (https://evidenceontology.org/annotation_resources/) and GitHub (https://github.com/evidenceontology/evidenceontology/blob/master/sri_eco_mappings.tsv).

## WEBSITE UPDATES

**Figure 3.**
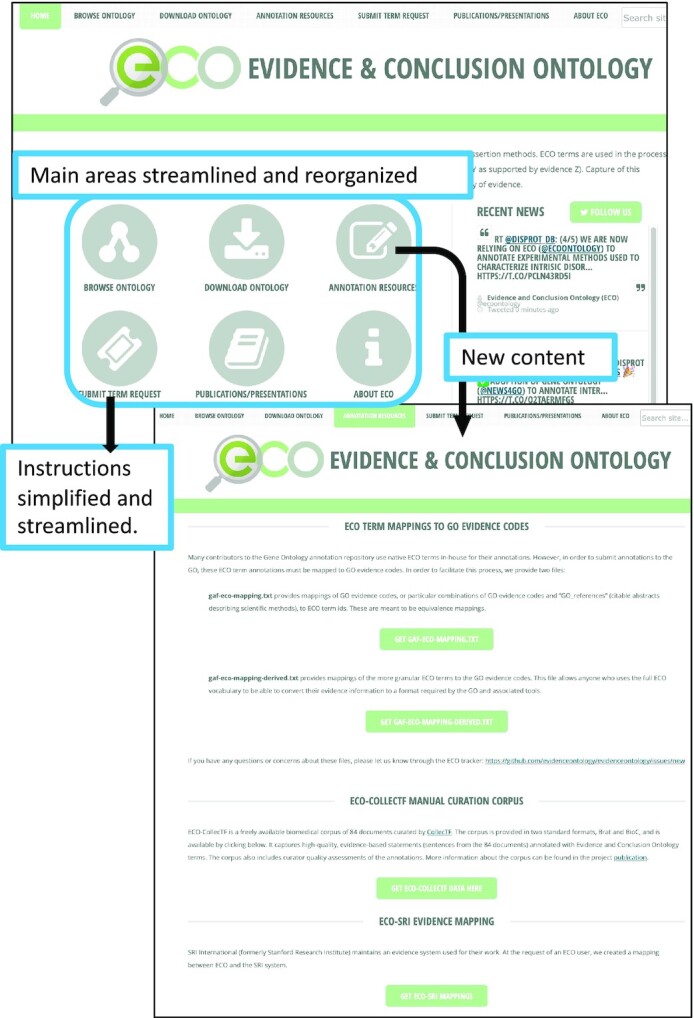
ECO website updates. This figure highlights some of the changes to the ECO website.

Since our last ECO update publication (2) we have done a general overhaul of the ECO website (Figure 3). We reorganized the content of the main areas (linked from the home page) to be more streamlined and intuitive. In addition, we added a new area (also linked from the home page) for ‘Annotation Resources’ which provides information about, and links to, the user resources described above, including the GO evidence code mapping files, ECO-CollecTF corpus, and ECO-SRI mappings. We have also streamlined the new term request guidelines linked from the home page and reorganized key information on publications. The ECO website is located at https://evidenceontology.org/.

## ECO RELEASES AND AVAILABILITY

New releases of ECO are made on a generally monthly schedule. ECO is completely freely available and is accessible through the ECO website (https://evidenceontology.org/) and GitHub (https://github.com/evidenceontology/evidenceontology). ECO is released into the public domain under a CC0 1.0 Universal license.

## FUTURE DIRECTIONS

### Continuing development and refinement of ECO

The ECO team will continue to work on development of ECO both in response to user requests and based on our continuing review and refinement of the ontology. We will continue our ongoing collaborations with multiple partners including the Gene Ontology, CollecTF, DisProt, the Ontology of Microbial Phenotypes, and many more. In addition, there are some specific areas of focused development that we plan to engage in. These are described below.

### Protein Ensemble Database (PED)

Our collaboration with DisProt has led to an additional collaboration with the Protein Ensemble Database (PED). PED is a deposition database of structural ensembles of intrinsically disordered proteins (IDPs). PED aims to capture the structural range that an IDP may encompass through the curation of representative sets of conformers that reflect the structural dynamics of a given IDP (23). Like DisProt, the PED has also decided to adopt ECO for capture of evidence information. We are now at the very beginning of our work on this project, with PED curators assembling an initial list of needed terms.

### Increase the use of logical axioms throughout ECO

As described above, all of the leaf nodes in ECO that include both evidence type and assertion method have logical axioms defining those relationships. In addition, nearly 200 ECO terms have logical definitions that include links to ontologies outside ECO such as OBI and GO. We plan to begin work to include logical definitions for additional (eventually all) ECO terms with an initial focus on the terms that currently have dual parentage. Per community guidelines for best-practices in ontology development (https://www.nist.gov/system/files/documents/2019/05/30/nist-ai-rfi-cubrc_inc_002.pdf)(24), it is best for terms to have just one asserted parent and to use logical axioms to allow additional parentage to be inferred by ontology reasoners. We will establish equivalence axioms as needed throughout ECO to provide the ability to assign additional axioms to all terms that currently have dual parentage.

## DATA AVAILABILITY

ECO is freely available from GitHub (https://github.com/evidenceontology/) and the project's website (http://evidenceontology.org/). ECO is released into the public domain under a CC0 1.0 Universal license.
